# Recent advances in stem cell-based therapies for type 1 diabetes: A glimpse into the future

**DOI:** 10.17305/bb.2025.12222

**Published:** 2025-03-19

**Authors:** Ahmed Hassanein, Saghir Akhtar

**Affiliations:** 1 College of Medicine, QU Health, Qatar University, Doha, Qatar

**Keywords:** Type 1 diabetes mellitus, T1DM, stem cells, encapsulation, immunosuppression, pancreatic beta cells

## Abstract

Type 1 diabetes mellitus (T1DM) is a serious, chronic metabolic and autoimmune disease that affects millions globally. While insulin administration remains the most effective treatment, it is not a cure. Long-term therapies, such as immunotherapy, can be effective for some patients, but they have notable limitations and do not provide a permanent solution. As a result, current research has shifted towards stem cell-based therapies, which offer a potentially expandable and scalable source of pancreatic beta cells. These therapies aim to restore long-term endogenous β-cell function in all T1DM patients, provided they can avoid immune recognition and rejection by the host. In this review, we will discuss the latest first-in-human successes of stem cell therapies for T1DM. We will then explore stem cell-derived islet encapsulation technologies and hypoimmune stem cells, examining how they might overcome the need for immunosuppressive therapy. Additionally, we will provide a summary of recent and ongoing biopharmaceutical industry pipelines and clinical trials for stem cell therapies aimed at treating T1DM. These advances suggest that stem cell therapies offer a promising and highly effective approach to treating patients with this chronic disease. However, large-scale clinical trials over the long term are necessary to verify these early successes and assess the curative potential of stem cell therapy for T1DM.

## Introduction

The International Diabetes Federation (IDF) estimates that nine million people worldwide currently have Type 1 diabetes mellitus (T1DM), a number projected to rise to 17.43 million by 2040 [1]. T1DM is a chronic autoimmune disease in which the body’s immune system destroys pancreatic beta cells, which produce insulin [2, 3]. This T cell-mediated destruction impairs insulin production, disrupting glycemic control and normal blood glucose levels [4, 5]. Uncontrolled blood sugar levels can lead to severe macrovascular and microvascular complications, including peripheral neuropathy, cardiovascular disease, and kidney damage, which may be life-threatening [6–9]. T1DM development is influenced by both genetic and environmental factors [10]. Among genetic factors, the HLA-DR3 and HLA-DR4 alleles are strongly associated with an increased risk of T1DM [3, 10, 11]. Environmental contributors include viral infections, reduced vitamin D levels, and altered gut microbiota [3, 12]. Recent efforts to prevent or delay beta cell destruction have led to antibody-based immunotherapy [13]. In 2022, the FDA approved Teplizumab (Tzield), an anti-CD3 monoclonal antibody, based on pivotal data from the TrialNet clinical trial (NCT01030861) and its extension study (NCT04270942, TN-10 Extension) [14]. These trials demonstrated Teplizumab’s safety and effectiveness [15, 16]. When administered as a single 14-day course of daily intravenous infusions, Teplizumab delayed the onset of stage 3 (clinical) T1DM by up to two years in high-risk, antibody-positive individuals with stage 2 T1DM [15], likely by attenuating T cell-mediated beta cell destruction. However, Teplizumab’s efficacy is limited to a subset of patients with early-stage (stage 2) T1DM, and it is associated with several adverse effects, including an increased risk of infections, cytokine release syndrome, hypersensitivity reactions, and lymphopenia [17].

For patients who develop T1DM, the current standard of care is insulin therapy [5]. However, the need for repeated insulin injections and continuous blood glucose monitoring imposes a significant financial burden and negatively impacts quality of life [5, 18]. Additionally, insulin therapy is a lifelong management approach rather than a cure [19]. It still requires frequent adjustments by both patients and healthcare providers, and the risk of hypoglycemia remains a concern [5]. To address these challenges, an ideal treatment would be an “artificial pancreas” or pancreatic beta cell therapy capable of automatically releasing insulin in response to blood glucose levels without triggering immune rejection. One recent advancement in this field is Lantidra (Cell Trans Inc. USA), an FDA-approved allogeneic pancreatic islet cell therapy. Lantidra involves isolating islet cells from a single deceased donor pancreas and infusing them into the hepatic portal vein to stimulate endogenous insulin secretion. In a clinical trial, 21 out of 30 T1DM patients who received Lantidra maintained insulin independence for a year, with 10 remaining insulin-free for over five years—demonstrating the potential of pancreatic beta-cell replacement therapy as a long-term treatment for T1DM [20]. Currently, Lantidra is indicated only for severely affected T1DM patients who experience frequent hypoglycemic episodes and struggle to achieve target HbA1c levels [21]. While it provides good glycemic control and reduces hypoglycemic risk, limitations include the need for lifelong immunosuppressive therapy, surgery-related complications, such as portal vein thrombosis and bleeding, and concerns over long-term efficacy, as some patients eventually required insulin again [22]. This relapse suggests that the number of transplanted islets may be insufficient to fully meet insulin demands, potentially necessitating repeated transplants. However, the scarcity of donor pancreases remains a major obstacle, highlighting the need for scalable alternatives—such as *in vitro* production methods that generate abundant islet cell progenitors or fully functional beta cells for broader accessibility [23]. As a result, current research is shifting toward stem cell-based therapies, which offer an expandable source of pancreatic beta cells to restore endogenous insulin production in all T1DM patients—provided they can be protected from autoimmune attack and immune rejection [24]. In this review, we will discuss the latest first-in-human successes of stem cell therapies for T1DM. We will then examine stem cell-derived islet encapsulation technologies and hypoimmune stem cells, which may eliminate the need for immunosuppressive therapy. Finally, we will summarize recent and ongoing biopharmaceutical industry pipelines and clinical trials investigating stem cell therapies for T1DM.

## A breakthrough clinical success in stem cell therapy for T1DM

Stem cell therapies hold significant potential for restoring or replacing the function of damaged beta cells in T1DM patients. Current approaches include the use of embryonic stem cells [25], xenogeneic sources [26], and autologous stem cells derived from a patient’s own tissues (e.g., adipose tissue) [27]. The latter approach, which is considered a more ethically acceptable alternative to embryonic stem cells, may also reduce the risk of immune rejection and has been gaining increasing interest [27]. The success of these therapies depends not only on the type of stem cells used but also on multiple other factors, including the choice of implantation site—whether in the liver, pancreas, subcutaneous tissue, or omentum [28–30]. The implantation site has been shown to influence the survival rate of beta cells, their integration into surrounding tissue, and immunocompatibility. Notably, a recent human clinical trial reported remarkable outcomes following the direct administration of stem cell-derived islets beneath the anterior rectus abdominal sheath [27].

### A ground-breaking clinical success in reversing T1DM using stem cells

**Figure 1.**
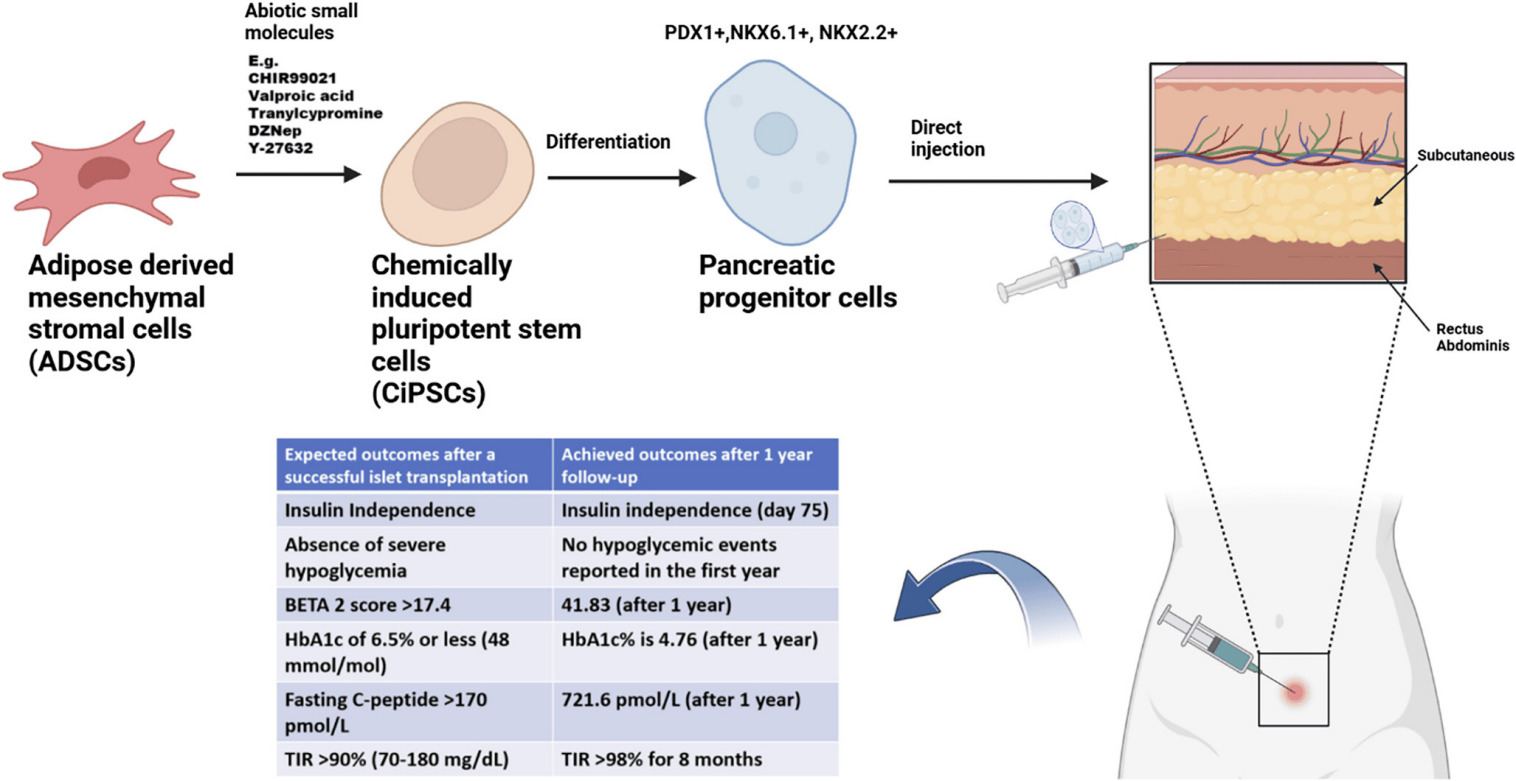
**A summary of the protocol in ChiCTR2300072200 and the preliminary results from the single female T1DM patient receiving CiPSCs in the trial.** ADSCs were extracted from the patient’s adipose tissue and reprogrammed into CiPSCs using small molecules targeting key signaling pathways (e.g., CHIR99021, valproic acid, tranylcypromine, DZNep, Y-27632) to enhance reprogramming efficiency. CiPSCs were then differentiated into pancreatic progenitor cells, confirmed by markers (PDX1+, NKX6.1+, NKX2.2+), and injected into the patient’s anterior rectus sheath. Glycemic control and graft success were monitored over a year, comparing expected vs achieved outcomes post-islet transplant. BETA 2 score provides a calculated estimate of islet graft function following islet transplantation based on the recipient's fasting glucose, fasting C-peptide, insulin dose and HbA1c. It is expressed as a single value (0 to 42); with higher scores associated with better graft function. Abbreviations: CiPSC: Chemically induced pluripotent stem cell; ADSC: Adipose-derived mesenchymal stromal cell; T1DM: Type 1 diabetes mellitus.

A groundbreaking clinical achievement in T1DM was recently reported: the world’s first successful direct injection of chemically reprogrammed autologous stem cells under the rectus abdominal sheath of a 25-year-old female patient receiving immunosuppressive therapy [27]. Following transplantation, the patient achieved insulin independence by day 75, maintaining it throughout a one-year follow-up period without any reported hypoglycemic events. This preliminary data is part of an ongoing Phase 1 clinical trial (ChiCTR2300072200) involving two additional T1DM patients. Given the significance of this breakthrough, a review of the study’s rationale and protocol is warranted. The study protocol involved isolating adipose-derived mesenchymal stromal cells (AD-MSCs) from the patient’s adipose tissue, which were then chemically reprogrammed *ex vivo* into chemically induced pluripotent stem cells (CiPSCs) [31]. CiPSCs are a type of pluripotent stem cell generated through chemical reprogramming, circumventing the ethical concerns associated with embryonic stem cells. Unlike conventional methods that rely on Yamanaka factors (c-Myc, Oct3/4, Sox2, and Klf4)—some of which have oncogenic potential—this approach utilized small abiotic molecules, significantly enhancing reprogramming quality and efficiency [27, 32–37]. For instance, valproic acid (VPA), one such molecule, increased reprogramming efficiency by 100-fold compared to transcription factor-based methods [38, 39]. Additionally, small abiotic molecules eliminate the need for sophisticated delivery technologies like viral vectors, which can introduce undesirable biological effects [40–44] and contribute to graft failure. In this study [27], the reprogrammed stem cells were induced to differentiate into pancreatic progenitor cells. This process was monitored by detecting key pancreatic progenitor cell-specific transcription factors, including PDX1, NKX6.1, and NKX2.2. The presence of these biomarkers confirmed successful differentiation, with the potential for further maturation into insulin-producing beta cells. The resulting CiPSC-islets consisted of 51% beta cells, 9% alpha-like cells, 8% delta-like cells, and 3% epsilon-like cells. These were implanted into the subanterior rectus sheath—an implantation site selected based on previous research demonstrating its superiority over hepatic and extrahepatic routes [45, 46]. A schematic summary of the trial protocol and clinical outcomes for this patient is presented in Figure 1.

The patient demonstrated marked glycemic control, with significant improvements in glycated hemoglobin (HbA1c), time in range (TIR), and fasting C-peptide levels. HbA1c decreased from a baseline of 7.57%–4.76% one year post-transplantation, well below the standardized success threshold of >7% [27, 47]. TIR improved substantially from 43.18% at baseline to over 98% at one year [27]. Additionally, fasting C-peptide levels surged from 0 pmol/L to 721.6 pmol/L, exceeding the normal range for healthy non-diabetics (300–600 pmol/L) [48]. Safety assessments, including tumor biomarkers (β-HCG, AFP, CEA, CA125, CA15-3, and CA19-9) and MRI imaging, found no evidence of tumorigenicity [27]. This clinical success with reprogrammed stem cell therapy was guided by findings from two non-human primate studies [45, 46]. The first study, which involved intraportal vein infusion of CiPSC-islets in rhesus macaques, showed initial glycemic improvements but was hindered by immune rejection, graft failure, and immunosuppression-related complications, leading to mortality [45]. The second study tested three implantation sites—the subanterior rectus sheath, brachioradialis muscle, and subcutaneous tissue—identifying the subanterior rectus sheath as the most effective. This site preserved islet volume and β-cell integrity while enhancing glycemic control and C-peptide levels [46]. Despite limitations, such as immune reactions, short observation periods, and the need for improved differentiation protocols, these studies laid the groundwork for the clinical trial [45, 46]. While this trial has yielded promising results, it has been conducted in only a single T1DM patient [27]. Expanding the study to a larger cohort and extending follow-up beyond one year are essential to fully assess the long-term safety and efficacy of stem cell therapy in T1DM. Furthermore, since this success was achieved with immunosuppressive therapy, additional studies are needed to determine whether similar outcomes can be attained without immunosuppression, which carries significant risks, particularly in T1DM patients.

Several key considerations regarding immunosuppression in this first-in-human clinical trial warrant attention. Firstly, immunosuppression masked the patient’s immune response to the autologous graft. Ideally, an autologous graft should not trigger graft-versus-host disease, unlike an allogeneic graft [49]. However, reprogramming stem cells and altering their genetic composition may affect their immunogenicity. If the reprogrammed cells express foreign antigens or modified markers, the immune system could recognize them as foreign [50]. In this trial, immunosuppression prevented the evaluation of the autologous CiPSC-islets’ immunogenicity, highlighting the need for further research in patients without immunosuppression. Secondly, because T1DM is an autoimmune disease, there is a risk that the host’s immune system could attack the autologous stem cells—just as it originally destroyed pancreatic beta cells [51]. This suggests that even use of modified stem cells with reduced immunogenicity might still require concomitant immunosuppressive therapy to prevent rejection. Finally, while immunosuppression may be acceptable for severe or critically ill T1DM patients who experience frequent hypoglycemic events and reduced quality of life, it is unlikely to be suitable for otherwise healthy T1DM patients. Long-term immunosuppression carries risks, such as opportunistic infections, malignancies, and other drug-related toxicities [52, 53]. Indeed, in ChiCTR2300072200, the immunosuppressed patient developed a severe upper respiratory tract infection requiring hospitalization within the first year of follow-up. To address the challenges associated with immunosuppression in T1DM patients, researchers are exploring protective cell encapsulation technologies and engineered hypoimmune stem cells as potential alternatives.

**Figure 2.**
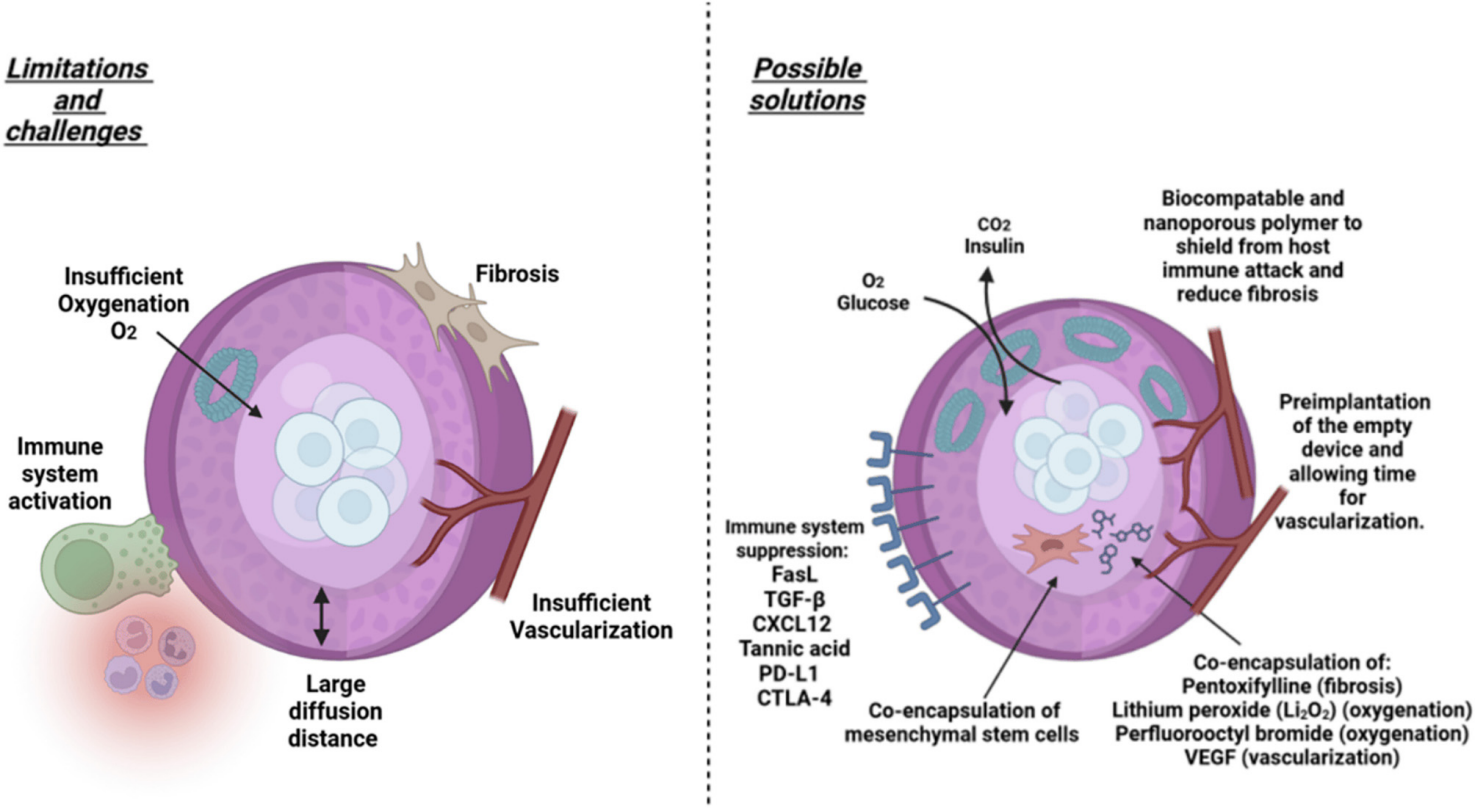
**Current limitations of encapsulation technologies (left hand panel) and possible solutions to mitigate them (right hand panel)**. Stem cell-derived islet encapsulation aims to shield islets from the host’s immune system using biocompatible/nanoporous biomaterials and immunomodulatory molecules (e.g., FasL, TGF-β, CXCL12, PD-L1). Gaseous and nutrient exchange is improved by increasing porosity and adding oxygen-liberating molecules (e.g., Li_2_O_2_, perfluorocarbons). Vascularization can be enhanced with VEGF or preimplantation of the empty device. Fibrosis is reduced by selecting biocompatible nanomaterials or incorporating anti-fibrotic agents (e.g., pentoxifylline). Diffusion distance is managed by optimizing capsule size and porosity (see main text for further details). Abbreviation: VEGF: Vascular endothelial growth factor.

## Islet cell encapsulation technology and hypoimmune stem cells

Developing an ideal stem cell therapy for T1DM without requiring immunosuppression involves two key steps. First, a robust differentiation protocol is needed to guide multipotent or pluripotent stem cells into pancreatic endocrine cells, ultimately generating functional insulin-producing beta cells. Second, protecting the transplanted islets from the host immune system can be achieved through advanced encapsulation technologies (e.g., bioartificial pancreases) or by engineering hypoimmune stem cells through genetic modifications. Each approach is discussed below.

### Islet cell encapsulation devices to avoid host immune recognition

Recent advancements in encapsulation technologies aim to eliminate the need for immunosuppression in islet transplantation by physically shielding islets from the host immune system (see Figure 2). The field has benefited from progress in nanomaterials and nanotechnologies that enhance the delivery of small-molecule drugs and biologicals, including cells [40, 41, 43, 54–56]. Stem cell-derived islet encapsulation designs should achieve several key goals. First, the islets must be coated with a biocompatible, protective membrane that shields them from immune attack. Second, this membrane should be semi-permeable to support cell survival by allowing adequate blood flow and exchange of oxygen, carbon dioxide, and nutrients [57]. Third, insulin secreted by beta cells within the encapsulation device must be released efficiently into the biological milieu. Fourth, the device must have sufficient cell-loading capacity to encapsulate an adequate number of stem cell-derived islets to meet physiological insulin demands, including postprandially [58]. Finally, the device should be fully retrievable in case of adverse biological effects. Islet encapsulation utilizes different size ranges, including nano- (<100 nm thickness), micro- (500–1000 µm diameter), and macro-encapsulation (≥1 mm diameter) [59, 60]. Device size determines the number of encapsulated stem cells and their implantation feasibility. Nano- and microencapsulation typically involve coating individual islets or small clusters of islet equivalents (IEQs) with a semi-permeable membrane, while macroencapsulation devices house hundreds to thousands of islets or IEQs. Studies suggest that at least 500,000 IEQs are required to reverse T1DM in patients [61], which could be achieved using a single microdevice or multiple nano- and micro-encapsulated devices. Device size also impacts nutrient and gas diffusion. Nanoencapsulation enables efficient exchange due to shorter diffusion distances but has a low cell-loading capacity. In contrast, macroencapsulation allows for a larger number of cells but is hindered by increased diffusion distances, which may compromise oxygen and nutrient supply, affecting cell viability [62]. Thus, beyond size and diffusion limitations, key challenges in achieving optimal therapeutic outcomes with encapsulation devices include immune rejection, fibrosis, oxygenation, and vascularization (see Figure 2).

**Table 1:** Strategies to mitigate key challenges in encapsulated stem cell therapy

**Challenge**	**Current limitation**	**Possible solutions**	**References**
Immune rejection and inflammation	White blood cells can infiltrate into the device and attack the pancreatic cells. Even if the membrane is impermeable to cells, antigens can enter and mount an immune response	Immunomodulatory cytokines and chemokines e.g., TGF-β and CXCL12 PEGylation with Polyethylene glycol Tannic acid antioxidant/PD-L1/FasL/CTLA-4	[77–81]
Fibrosis	Fibrotic tissue can grow over the encapsulated device, diminishing its blood, oxygen and nutrient supply and impeding its therapeutic effects	Co-transplantation of mesenchymal cells which has shown to decrease fibrosis, Hyaluronic Acid/Collagen Hydrogel, loading anti-fibrotic drugs like pentoxifylline into the microspheres, using ultra-purified alginate	[67, 87, 88, 171]
Oxygenation	Insufficient oxygenation hinders survival of the stem cells leading to hypoxia of the islets and ultimately to graft’s failure	Biomimetic scaffolds that improve internal structure to enhance oxygen delivery, in situ oxygen production using lithium peroxide (Li_2_O_2_), adding Perfluorocarbons to the encapsulating material or loading the device with Perfluorocarbons e.g., alginate hydrogel enriched by PFOB	[89, 91, 92]
Vascularization	Islets are highly vascularized and grafts usually fail due to inadequate vascularization and blood supply	Pre-vascularization of the device and loading the encapsulation device with VEGF are possible solutions	[94, 95]
Large diffusion distance	Insulin has longer distance to travel which renders it from exerting its therapeutic effects, also nutrients and oxygen take longer to reach the islets which leads to hypoxia	Pressure-driven flow (macroencapsulation) Optimize geometry (microencapsulation) Nanoporous membranes	[96–99]

A wide range of nanomaterials have been explored for their biocompatibility in islet cell encapsulation, including natural polymers, such as chitosan, poly-L-lysine, alginate, and collagen, as well as synthetic and semisynthetic materials like polyethylene glycol, polyvinyl alcohol, polyacrylamide, and Teflon (polytetrafluoroethylene, PTFE) [60, 62]. Among these, alginate is one of the most widely studied biomaterials in stem cell therapy due to its exceptional biocompatibility, non-toxicity, and ease of preparation [63]. Alginate is a natural polysaccharide derived from brown seaweed, composed of β-D-mannuronic acid (M units) and α-L-guluronic acid (G units) [64]. In the presence of divalent cations, such as Ca^2^^+^ or Ba^2^^+^, alginate forms ionic cross-links, a gelation property that enables the creation of alginate microspheres or microgels for embedding pancreatic islets [65, 66]. These semi-permeable, biocompatible microspheres allow the exchange of low-molecular-weight nutrients, gases, and insulin (∼5.8 kDa) while blocking larger molecules (>150 kDa), such as antibodies. This barrier prevents encapsulated cells from being recognized as foreign by the host’s immune system [60, 67]. Alginate microspheres have shown significant promise in supporting islet cell viability and function. *In vitro* studies have demonstrated prolonged C-peptide secretion from alginate-encapsulated islets for over 75 days in most cases, with some studies reporting viability up to 180 days [68]. Similarly, in diabetic rats, subcutaneous administration of islet cells encapsulated in alginate microcapsules coated with poly[2-(methacryloyloxy) ethyl] trimethylammonium chloride (PMETAC), a biocompatible semi-permeable membrane, maintained long-term cell viability and insulin production for up to 90 days—compared to just seven days for non-encapsulated islets [69]. These findings suggest the potential for a long-term functioning device with clinical applications. However, the presence of impurities in alginate biopolymers has led to immune reactions *in vivo* [70]. Recent advancements in ultra-purified alginate have significantly reduced its immunogenicity by eliminating contaminants, such as lipopolysaccharides, lipoteichoic acids, and peptidoglycans, which activate Toll-like receptors and trigger immune responses [70, 71]. High-purity formulations, such as UP-LVG and UP-MVG, which contain at least 60% guluronate monomers, have demonstrated superior biocompatibility and improved *in vivo* performance in transplantation models [72, 73]. Additionally, the ratio of M and G units in alginate plays a crucial role in determining its immunogenicity, with higher G-unit formulations inducing significantly lower immune responses than those with a higher M-unit content [74]. Functionalizing alginate with bioactive molecules has further improved graft survival and integration. For example, incorporating the RGD (Arginylglycylaspartic acid) motif, which interacts with integrins to enhance cell adhesion [75], or VEGF to promote local vascularization [76], has led to better outcomes. Therefore, careful optimization of alginate composition, purification processes, and functionalization strategies is essential for minimizing chronic inflammation, reducing fibrosis, enhancing graft survival, and improving the therapeutic efficacy of encapsulated stem cells.

Despite significant advancements in encapsulation technologies, further research is needed to address five key challenges that hinder optimal therapeutic outcomes: immune rejection, fibrosis, oxygenation, vascularization, and diffusion distance (see Table 1 and Figure 2). Current encapsulation methods require refinement to evade immune detection and promote immune tolerance. Strategies such as incorporating immunomodulatory molecules—including FasL, TGF-β, CXCL12, tannic acid, polyethylene glycol and immune checkpoint inhibitors like PD-L1 and CTLA-4—have shown potential in fostering a tolerogenic environment and preventing immune rejection [77–82]. Fibrosis remains a major barrier to the long-term survival of both encapsulated islets and encapsulation devices [83]. This process, driven by macrophages and fibroblasts, leads to the formation of a dense extracellular matrix that creates a hypoxic environment, exacerbating β-cell dysfunction and impairing insulin secretion [83]. Various strategies have been explored to mitigate fibrosis, including the use of ultra-purified alginate to reduce immune activation [67], co-encapsulation with mesenchymal stem cells (MSCs) (which release anti-fibrotic factors) [75, 84], and the incorporation of immunomodulatory molecules, such as TGF-β and CXCL12 to promote local immune tolerance [85, 86]. Additional approaches include using hyaluronic acid/collagen hydrogels and incorporating controlled-release anti-fibrotic agents like pentoxifylline [87, 88]. While these methods have shown promise in rodent and small-animal models, further validation in non-human primates and clinical trials is essential to determine their effectiveness in preventing fibrosis and improving therapeutic outcomes in human patients. A major challenge in encapsulation therapy for T1DM is insufficient oxygen delivery to encapsulated islets, as most devices do not support intra-device vasculature. This can lead to hypoxia and cell death, significantly limiting therapeutic success. To address this, a biomimetic scaffold—Speedy Oxygenation Network for Islet Constructs (SONIC)—has been developed, inspired by insect tracheal gas exchange. This system integrates multiple internal continuous air channels within a hydrogel matrix, significantly enhancing oxygen delivery to encapsulated islet cells, even in large (6.6 mm thick) devices [89]. When implanted into the peritoneal cavity of a diabetic mouse model, the SONIC device maintained islet cell viability and functionality for up to six months [89].

Another biologically inspired study explored the incorporation of chloroplasts—plant organelles responsible for oxygen production—into alginate microcapsules [90]. Chloroplasts were chemically integrated into the alginate using a chloroplast-transit-peptide (CTP), a short amino acid sequence that directs and anchors proteins to the chloroplast. CTP not only enhanced chloroplast adhesion to the alginate biofilm but also upregulated key photosynthesis genes (e.g., psbA, psbO, rps12, rpl14), boosting chloroplast-mediated oxygen synthesis even under hypoxic conditions (∼1% pO_2_). These CTP-alginate microcapsules, known as the respiratoid biosystem, were loaded with cadaveric pancreatic islets and successfully sustained oxygenation for at least 85 days following intraperitoneal transplantation into a diabetic mouse model [90]. These findings highlight the potential of nature-derived biomimetic strategies in addressing the oxygenation challenge in future encapsulation devices. In situ oxygen production within a cell-encapsulation device is another promising strategy for improving stem cell oxygenation. For instance, a chamber containing lithium peroxide (Li_2_O_2_) can be placed within the encapsulation device, where it reacts with CO_2_ produced by stem cells to generate oxygen in a process known as inverse breathing [91]. Additionally, perfluorocarbons—compounds with a high capacity for dissolving oxygen—can significantly enhance oxygenation and cell survival when either incorporated into the encapsulation material (e.g., perfluorooctyl bromide [PFOB]) or loaded into a separate chamber [92]. Vascularization and diffusion distance limitations present further challenges to the success of encapsulated islet therapy. One potential solution is the preimplantation of an empty device, allowing time for vascularization to occur before introducing stem cell-derived islets. This two-step approach has been successfully implemented in the Sernova Cell Pouch, where cell viability was maintained for up to five years in T1DM patients [93, 94]. While these results are based on cadaveric islets, Sernova, in collaboration with Evotec, aims to integrate iPSC-derived islets into their delivery system, which could expand the applicability of this technology. Moreover, co-encapsulation of pro-angiogenic factors such as vascular endothelial growth factor (VEGF) can promote local angiogenesis at the implantation site, further enhancing nutrient and gas exchange as well as islet cell viability and functionality [95]. Finally, addressing the long diffusion distances in macroencapsulation devices—commonly used clinically due to their high cell-loading capacity—may be possible by applying modest pressure to facilitate insulin efflux, rather than relying solely on passive diffusion [96]. Optimizing the geometry of microencapsulation devices can also help maximize surface area and improve insulin diffusion [97]. Additionally, careful selection of single or multiple nanoporous membranes could optimize the diffusion of insulin, oxygen, and essential nutrients such as glucose [98, 99]. Further research into these optimization strategies will be crucial for enhancing the long-term immune protection of stem cell-derived islets, improving cell viability, and sustaining insulin release from encapsulation devices. While many of these approaches have shown promise in preclinical models, clinical trials will be essential to evaluate their real-world applicability.

### Hypoimmune stem cells

Hypoimmune stem cells are advanced genetically engineered cells designed to evade the immune system and prevent graft rejection [100]. Typically, gene editing technology is used to modify expression of immunoregulatory genes so as to either downregulate immune-activating markers (e.g., Class I and Class II HLA molecules, costimulatory molecules [CD80 and CD86], MHC Class II molecules, Toll-like receptors, or complement-activating proteins), and/or to upregulate immune-inhibitory markers (e.g., HLA-G, PD-L1, PD-L2, and CD47 that typically send “don’t eat me” signals to macrophages [101, 102]). Thus, by combining the downregulation and the (over-) expression of these different markers, hypoimmune stem cells that can evade both the adaptive and the innate immune system can be produced and offer a promising solution for transplantation without the need immunosuppressive therapy. Such an approach has been employed in an ongoing clinical trial using Sana Biotechnology’s proprietary Hypoimmune Platform (HIP) whereby cells have been engineered to avoid immune rejection through (a) CRISPR-Cas9-mediated inactivation of the immune-activating B2M and CIITA genes, and (b) lentiviral-mediated overexpression of CD47 [103, 104].

Preclinical studies in humanized mice and non-human primates have demonstrated that engineered islets (B2M-/-, CIITA-/-, CD47+), whether cadaveric or derived from human iPSCs, exhibited reduced expression of HLA class 1 and II molecules and were reported to successfully avoid immune detection and macrophage-mediated phagocytosis whilst maintaining sufficient insulin production for glycemic control [103–105]. In a study utilizing human cadaveric islets, intramuscular implantation of HIP-engineered cadaveric islets in a humanized mouse model resulted in significant glycemic control during the study period of one month [103]. By day 29 post-transplant, blood glucose levels reached below 400 mg/dL following a glucose challenge test and the mice exhibited a concomitant mean C-peptide level of 1700 pmol/L indicating marked insulin production [103]. Similar results were obtained with HIP technology in a non-human primate. In a primate model of T1DM, implantation of cadaveric monkey islets intramuscularly into a single cynomolgus monkey normalized C-peptide levels one week after the transplant and remained stable for up to six months. The primate became insulin independent and required no exogenous insulin after two weeks. Serum analysis indicated no cellular and antibody-mediated killing of the engineered islets indicative of successful immune protection. A notable safety feature of this technology is the possibility of using an anti-CD47 antibody that neutralizes the overexpressed CD47 and can facilitate the removal the implanted hypoimmune stem cells by the host’s natural immune system should toxicities and uncontrolled division occur over time [103, 105].

In a study utilizing allogenic stem cell-derived islets, transplantation of HIP-iPSC-derived pancreatic islets into the thigh muscle of eight humanized diabetic mice demonstrated the engineered islets’ ability to maintain endocrine function and cell viability throughout a 30-day observation period. HIP-iPSC islet-transplanted mice demonstrated significant reduction in fasting blood glucose, reaching 200 mg/dL by day 28. Additionally, blood glucose levels were successfully maintained below 400 mg/dL on day 30 following oral glucose challenge test. Immune assays and histological examination confirmed HIP islets’ survival without immune infiltrations reflecting the potential of such a therapy in evading the host immune system and achieving considerable glycemic control similar to that obtained with cadaveric islets [104].

Based on these preclinical findings, Sana Biotechnology currently has two hypoimmune cell technologies in preclinical development for T1DM; UP421 (using modified cadaveric pancreatic islets) and SC451 (stem cell-derived pancreatic islets) [106]. The results of these clinical studies are much awaited. While hypoimmune stem cells hold great promise, it is important to note their current limitations, including the potential for off-target effects from gene editing technologies like CRISPR-Cas9 and an increased risk of tumorigenicity due to unintended genetic alterations that may disrupt normal cellular function [107]. Thus, further research and clinical validation are clearly required to confirm their safety, efficacy, and long-term potential for use in humans.

## Key biopharmaceutical pipelines for stem cell therapies for T1DM

**Figure 3.**
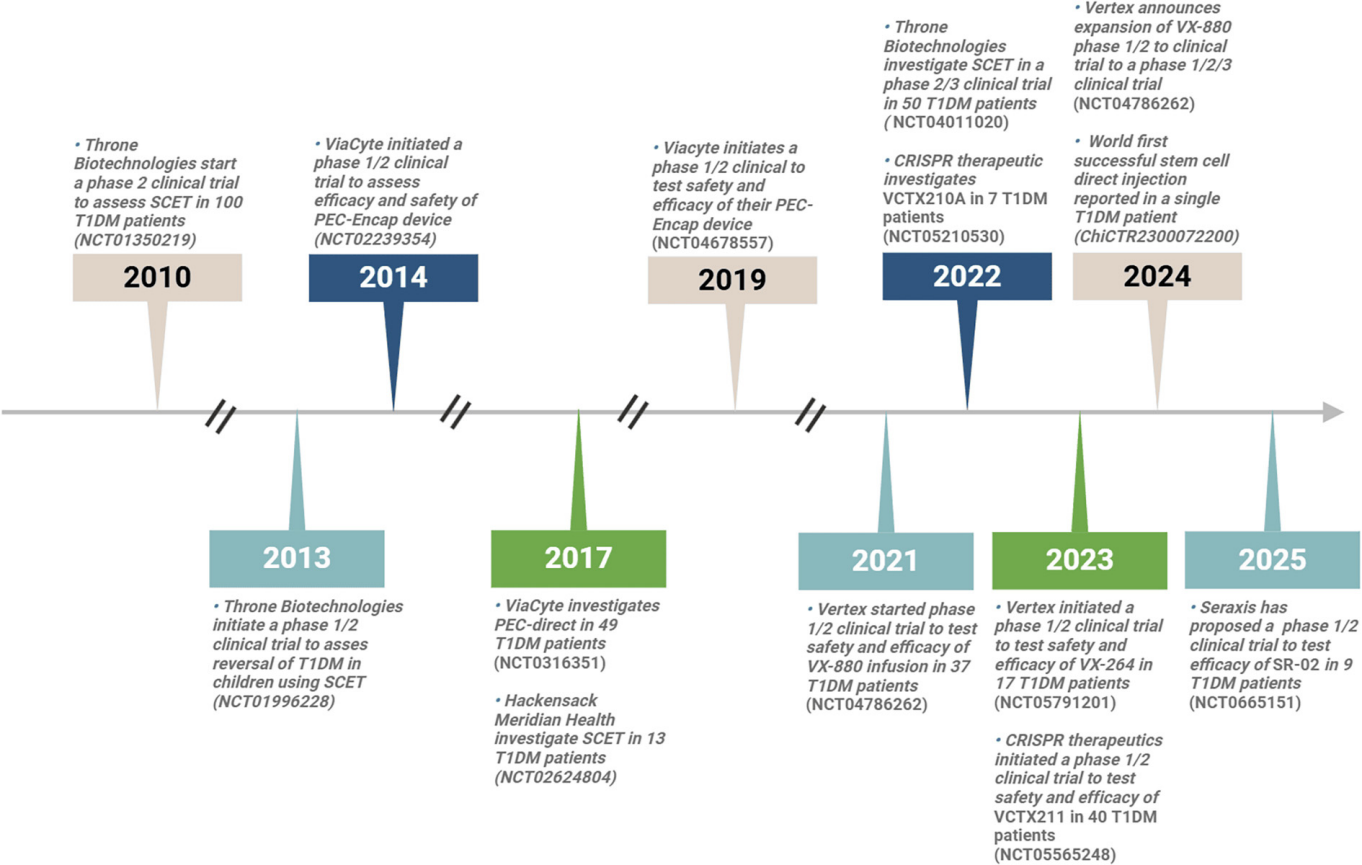
**Timeline of the different stem cell therapy clinical trials in T1DM patients over the last 15 years.** The figure reflects the accelerating clinical progress of stem cell therapies in T1DM. Abbreviation: T1DM: Type 1 diabetes mellitus.

Pluripotent stem cells can give rise to nearly all of the ∼200 cell types in the human body, whereas multipotent stem cells are restricted to differentiating into a specific range of cells within a particular lineage. Various pluripotent and multipotent stem cells have been extensively studied for their potential in treating T1DM. Several biotechnology and pharmaceutical companies, including Vertex, CRISPR Therapeutics, Seraxis, and Throne Biotechnologies, are actively conducting clinical trials to assess the safety and efficacy of stem cell-based therapies for T1DM (see Table 2 and Figure 3). Among multipotent stem cells, MSCs have shown promise in improving glycemic control in T1DM [108]. However, their clinical application is limited by several challenges, including functional variability that complicates consistency, the transient nature of their therapeutic effects, and the need for precise regulation of their immunomodulatory properties [109]. A comprehensive discussion of MSCs is beyond the scope of this review; for detailed insights into their applications and associated challenges in T1DM, readers are referred to existing reviews [109, 110]. Here, we focus on the pipelines of major biopharmaceutical companies developing non-MSC approaches.

### ViaCyte’s PEC-direct and PEC-encap: Challenges in vascularization and engraftment

ViaCyte, acquired by Vertex Pharmaceuticals in 2022, developed a series of stem cell-derived islet cell therapies, including PEC-Direct (VC-02) and PEC-Encap (VC-01). These therapies are based on pancreatic endoderm cells (PEC) derived from the CyT49 stem cell line, which are then loaded into delivery devices. The pancreatic endoderm serves as a precursor to both exocrine and endocrine pancreatic tissues, from which endocrine cells are harvested and placed into the PEC-Direct device. PEC-Direct features a multi-layered delivery structure consisting of (a) a semi-permeable membrane made of expanded polytetrafluoroethylene (ePTFE) with engineered openings and (b) an outer polyester mesh that provides rigidity and stability [111]. Unlike fully enclosed encapsulation devices, PEC-Direct allows direct vascularization between the islets and surrounding tissue, significantly increasing the risk of immune rejection. Unfortunately, results from a Phase 1/2 trial (NCT03163511) involving 10 patients showed limited clinical benefits. Only one patient experienced a modest improvement in TIR and C-peptide levels, with an insufficient beta cell mass of just 4%—far below the threshold for clinical efficacy [112–115] (see Table 2). This suboptimal outcome may be attributed to the selection of a subcutaneous implantation site, which has been shown to have poor oxygen supply and inadequate vascularization [46]. Additionally, inherent limitations of ePTFE may have contributed to these challenges. While ePTFE is known for its biocompatibility, its inert nature hinders effective tissue integration, leading to limited vascularization [116] and an increased risk of graft rejection [117] (see Table 1 for possible solutions). Furthermore, the lack of transparency regarding ViaCyte’s ePTFE manufacturing processes raises concerns, as variations in membrane thickness and mechanical properties can hinder oxygen and nutrient diffusion, ultimately compromising therapeutic efficacy [118]. Surface topology and porosity also influence biointegration and immune response, with larger pore-sized ePTFE exhibiting reduced fibrosis [119]. These insights highlight the need to optimize ePTFE materials—both in surface properties and manufacturing processes—to improve graft success and long-term therapeutic outcomes. Beyond PEC-Direct, ViaCyte also developed PEC-Encap, a macroencapsulation device designed to prevent direct immune exposure while permitting vascularization on its surface without capillary penetration [120]. While this approach protects the stem cells from immune attack, it also limits nutrient, oxygen, and insulin diffusion, impairing islet function [120]. An initial Phase 1/2 trial (NCT02239354) failed due to poor engraftment and adverse effects, and a subsequent trial (NCT04678557) launched in 2020 showed minimal efficacy, with only one patient exhibiting detectable C-peptide increases [59, 121] (see Table 2). The clinical interim report lacked details about the device, making it difficult to pinpoint the exact reasons for its failure.

**Table 2:** A selection of recent clinical trials in encapsulation, non-encapsulation strategies, and stem cell educator therapy

	**Description/** **immunosuppression**	**Patients enrolled**	**Phase**	**Trial identifier/status**	**Results and outcomes**
*Encapsulation*					
PEC-Encap	PECs are loaded in an encapsulation device that is implanted subcutaneously/ no immunosuppression	19 T1DM patients **1^st^ cohort:** 19 patients, nine completed **2^nd^ cohort:** 0	Phase 1/2	NCT02239 354/ Terminated	**1^st^ cohort:** 2/19 developed serious AEs, 18/19 developed non-serious AEs **2^nd^ cohort:** was never initiated due to AEs and insufficient functional product engraftment
		31 T1DM patients **1^st^ cohort:** 17 patients, 16 completed **2^nd^ cohort:** 14 patients, 0 completed	Phase 1/2	NCT04678 557/ Terminated (2021)	**1^st^ cohort:** 1/17 had serious AEs, 17/17 developed non-serious AEs **2^nd^ cohort:** 3/14 had serious AEs, 14/14 developed non-serious AEs, zero completed due to lack of efficacy and safety concerns, only one subject showed detectable increase in C-peptide after MMTT, <0.1 ng/mL, which is not sufficient, Insufficient functional engraftment of device
VCTX211	Genetically modified PEC211 loaded into a perforated encapsulation device	40 T1DM patients	Phase 1/2	NCT05565 248/ Recruiting, completion by 2025	**NA**
**VX-264	Same VX-880 islets are encapsulated and implanted, no immunosuppression	17 T1DM patients	Phase 1/2	NCT05791 201/ Recruiting, completion by 2026	**NA**
*Non-encapsulation*					
PEC-direct	PECs are transplanted into an open scaffold made of ePTFE that allows direct vascularization, requires immunosuppression	49 T1DM patients **1^st^ cohort:** two patients, one completed **2^nd^ cohort:** 47 patients, 14 completed	Phase 1/2	NCT03163 511/ Completed	**1^st^ cohort:** No serious AEs, 2/2 showed non-serious AEs. **2^nd^ cohort:** 19/47 had serious AEs, 47/47 showed non-serious AEs. 17 patients: Insulin expression and engraftment were observed in 63% of PEC-direc t units. 6/17 subjects showed positive C-peptide as early as first six months after implant. 11/17 showed no detectable increase in circulating C-peptide after MMTT [111]. 15 patients: two patients withdraw, five others were recommended to withdraw at nine months due to undetectable C-peptide. Insulin requirements reduced by 20%, TIR increased by 13% only. Only one patient showed >50% reduction in insulin requirement, no patient achieved insulin independence after one year. four patients had >0.03 nmol/L C-peptide after MMTT which might have some clinical benefit but is only 1/10^th^ to 1/30^th^ of C-peptide levels required for insulin independence [172].
VCTX210 A	Genetically modified PEC210A to reduce immunogenicity loaded into a perforated device	Seven T1DM patients	Phase 1	NCT05210 530/ Completed	**NA**
SR-02	Allogeneic pancreatic endocrine cell clusters implanted in omentum, requires immunosuppression	nine T1DM patients	Phase 1/2	NCT06651 515 / Not yet recruiting completion by 2028	**NA**
Reprogrammed stem cells	Autologous stem cell-derived islets/ implanted subanterior rectus sheath	3 T1DM patients	Phase 1	[ChiCTR23 00072200] (https:// www.cell. com/cell/ abstract/ S0092-867 4(24)0102 2-5)/Recruiting,	1 patient: TIR increased from 43.18% to 96.21% by four months after transplan tation, HbA1c at 4.76% after one-year, Fasting C-peptide increased to 721.6 pmol/L at one year, Insulin independence reached by day 75
VX-880	Allogenic stem cell-derived and insulin producing islets are infused into hepatic portal vein necessitating chronic immunosuppression	37 T1DM patients, then expanded to enroll 50 patients	Phase 1/2 converted recently to phase 1/2/3 [129]	NCT04786 262/ Recruiting, completion by 2030	12 patients: All 12 patients enrolled showed islet cell engraftment, insulin production by day 90, >70% TIR, HbA1c levels <7.0%, and elimination of hypoglycemic events. 11/12 patients eliminate d or reduced exogenous insulin. 3/12 stopped exogenous insulin at 270-, 180-, and 180-days post-treatment and were considered insulin independent after maintaining positive outcome at the 12 months post-treatment follow up. 7/12 no longer needed exogenous insulin but not considere d insulin independe nt as they have not reached 12 months of follow up. The other two still in early stages post infusion. Majority of AEs are mild and moderate with no severe AEs [127, 169].
Stem cell educator therapy	Blood is drawn and immune cells are cultured with CB-SCs to modulate their autoimmunity	15 T1DM (single SCET treatment)	Phase 1/2	NCT01350 219/ Unknown status	15 patients: 12 received treatment, three received sham. Treatment group divided to group A (six patients with residual Beta cells) and group B (six patients without residual Beta cells). Group A and group B showed reduction in daily insulin doses by 38% and 25%, respectively. Improvement in fasting C-peptide and C-peptide levels post-OGTT for both groups, with larger increase for group A, even after 40 weeks of SCET [139].
		15 T1DM (two SCET treatments)	Phase 1/2		15 patients: All participant received two stem cell educator therapy treatments. Second treatment after three months. 56 weeks post treatment, both group A (patients with residual Beta cells) and group B (without residual cell function) showed similar fasting C-peptide levels, median HbA1c, and median daily doses of insulin to the baseline levels, reflecting SCET ability to rescue Beta cells from the typical decline in beta cell function in T1DM [140].
		50 T1DM patients	Phase 2/3	NCT04011 020/ Recruiting, completion by 2025	**NA**

** VX-264 has been discontinued as of March 28, 2025; see note added in proof at the end of manuscript. Abbreviation: T1DM: Type 1 diabetes mellitus; SCET: Stem cell educator therapy.

### CRISPR therapeutics and PEC-QT: Gene-editing for immune evasion

To address immune rejection, ViaCyte partnered with CRISPR Therapeutics in 2018 to develop PEC-QT (VCTX210), incorporating CRISPR/Cas9 gene editing to reduce the immunogenicity of PECs [122]. The VCTX210A device, derived from PEC-Direct, used modified PEC210A cells to evade immune system activation [123]. A Phase 1 trial (NCT05210530) was completed in 2023, though results have not yet been disclosed. Subsequently, a Phase 1/2 trial (NCT05565248) was launched to evaluate a modified version of the product, VCTX211, using PEC211 cells in 40 T1DM patients. The trial’s expansion suggests potential early efficacy signals, though data remains unavailable. In early 2024, Vertex Pharmaceuticals ended its collaboration with CRISPR Therapeutics, leaving CRISPR solely responsible for CTX211’s continued development. However, as of this writing, no further updates have been released.

### Vertex’s VX-880 and VX-264

Vertex Pharmaceuticals is a leader in regenerative medicine, advancing stem cell therapies for T1DM. The company has two stem cell therapy candidates in development for T1DM: VX-880 and VX-264. Its progress in this field was significantly accelerated by acquiring two biotech companies—Semma Therapeutics in 2019 and Viacyte in 2022.VX-880, formerly known as STx-02, is based on Semma Therapeutics’ proprietary technology, which uses allogenic pancreatic islet stem cells infused into the hepatic portal vein to restore endogenous insulin production. Preclinical studies in non-human primates and pigs showed promising results, including C-peptide secretion and improved glycemic control, both with and without immunosuppression [124, 125]. However, the VX-880 clinical trial was conducted with concomitant immunosuppressive therapy. In 2021, Vertex initiated a Phase 1/2 clinical trial (NCT04786262) to evaluate the safety and efficacy of VX-880 in 37 T1DM patients. Early results were encouraging, with three of six participants achieving insulin independence within 180–270 days [126]. Although the trial was temporarily paused in early 2024 due to two unrelated patient deaths, it resumed later that year. Updated data from 12 enrolled patients showed >70% time-in-range, HbA1c <7.0%, and significant reductions or complete elimination of exogenous insulin use [127]. The study has since expanded into a Phase 1/2/3 trial enrolling 50 patients to further assess long-term efficacy and safety [128] (see Table 2). In 2023, Vertex launched a Phase 1/2 clinical trial (NCT05791201) to evaluate VX-264, a stem cell therapy designed to eliminate the need for immunosuppression. This trial, expected to enroll 17 patients and conclude by 2026, could offer a promising alternative to current treatments. However, as no data are yet available, it is too early to assess the therapy’s efficacy or the performance of its device technology.

### Seraxis and SR-02

Seraxis is developing SR-02, a stem cell-derived therapy for T1DM, using pancreatic endocrine islets differentiated from its proprietary SR1423 stem cell line. The SR1423 line originates from allogeneic pancreatic cells isolated from a deceased donor’s pancreas, which are then reprogrammed into definitive endoderm tissue—a precursor to both endocrine and exocrine pancreatic cells [129]. This approach minimizes the risk of off-target differentiation and tumorigenesis [129]. Through a specialized differentiation protocol, high-purity islet-like clusters are generated, which self-assemble into organoids. These organoids are then loaded into Seraxis’s proprietary SeraGraft device—designed to protect the islets from immune rejection while promoting vascularization—though specific details about the device remain undisclosed [129, 130]. A Seraxis study in a mouse model revealed that pancreatic endocrine cells lacking the differentiation marker NKX6.1, but expressing the transcription factors ISL-1 and MAFA, secreted higher levels of C-peptide and maintained glycemic homeostasis longer than NKX6.1-positive cells [131]. This finding represents a paradigm shift in pancreatic progenitor cell differentiation, as NKX6.1 positivity was previously considered necessary for functional islet cell development [132, 133]. Despite similar *in vitro* potency, NKX6.1-negative cells demonstrated superior engraftment and function following omental transplantation in rodents. These findings challenge conventional beta cell differentiation protocols and contribute to the high purity of SR1423-derived islets [131]. Recently, Seraxis received FDA Investigational New Drug (IND) clearance to initiate a Phase 1/2 clinical trial (NCT06651515) to evaluate SR-02 in T1DM patients. The trial, expected to begin in early 2025, will enroll nine patients. While the SeraGraft device is designed for use in immunocompetent T1DM patients, immunosuppression will be employed in this initial trial to assess potential clinical effects.

### Throne biotechnologies and stem cell educator therapy

Cord blood-derived multipotent stem cells (CB-SCs) have shown significant potential in regenerative medicine, particularly for treating T1DM. One notable application is Throne Biotechnologies’ stem cell educator therapy (SCET), which leverages CB-SCs to modulate T cell activity and prevent autoimmune attacks on pancreatic beta cells [134]. Unlike other stem cell therapies that aim to differentiate stem cells into functional beta cells, SCET instead “educates” the patient’s T cells to develop immune tolerance, preventing them from attacking beta cells [135]. In SCET, a patient’s blood is processed through an apheresis machine to separate white blood cells, which are then passed through the SCET device. This device is coated with adherent CB-SCs that are firmly attached between a top covering plate and a bottom collecting plate, ensuring that no foreign stem cells enter the body [136]. CB-SCs help “educate” T cells by inducing immune tolerance through their expression of the autoimmune regulator (AIRE), which modulates T cell activity, and Galectin-9, which suppresses B lymphocytes. Additionally, CB-SCs secrete exosomes that polarize macrophages into their M2 anti-inflammatory phenotype [135]. SCET also enhances beta cell function by increasing the uptake of platelet mitochondria, promoting beta cell proliferation [137]. Together, these mechanisms protect pancreatic beta cells from autoimmune destruction and preserve their insulin-secreting ability, making SCET a promising therapy for T1DM. SCET offers several advantages: it has minimal to negligible side effects, introduces no foreign stem cells as it is an *ex vivo* method, and has low immunogenicity due to the absence of CD3+ T cells, eliminating the need for HLA genotyping [138]. Throne Biotechnologies launched a phase 1/2 clinical trial in 2010 (NCT01350219), which demonstrated that a single SCET treatment significantly improved C-peptide levels, reduced HbA1c, and lowered daily insulin requirements in T1DM patients [139]. The therapy increased regulatory T cells (Tregs) and helped restore the balance of Th1/Th2/Th3 cytokines. It was particularly effective in patients with residual beta cell function, suggesting greater benefits for early-stage T1DM. A subsequent cohort receiving two SCET treatments also showed sustained improvements in fasting C-peptide levels, HbA1c, and insulin dosage, further highlighting SCET’s potential for long-term T1DM management [140] (see Table 2).

A four-year follow-up of nine patients demonstrated promising safety outcomes, with no reported infections or tumor development. Two patients maintained normal fasting C-peptide levels, while one showed significant improvement, though not reaching normal levels. The remaining six experienced a decline in C-peptide levels, suggesting a potential need for booster treatments to sustain therapeutic benefits [137]. Throne Biotechnologies is now conducting a phase 2/3 trial (NCT04011020) with 50 patients, expected to conclude by June 2025, to further evaluate SCET’s safety and efficacy, with the possibility of FDA approval [136].

### Preclinical innovations: Selected examples

In addition to the clinical trials mentioned above, several approaches remain in the preclinical stage, with some showing strong potential to advance into clinical trials. Here, we discuss two examples: IsletRx-iTOL-100 and SIG-002, both of which have demonstrated encouraging preclinical results.

### IsletRx- iTOL-100 (Kadimastem and iTolerance)

The IsletRx-iTOL-100 (iTOL-102) combinational product, developed through a collaboration between Kadimastem and iTolerance, represents a promising preclinical advancement in islet cell transplantation. IsletRx is a tradename for pancreatic islets derived from embryonic stem cells, encapsulated using iTolerance’s proprietary microencapsulation technology, iTOL-100 [25]. This technology employs an SA-FasL microgel, composed of FasL-streptavidin and biotin-polyethylene glycol microgel. The FasL component targets the FasL receptor on activated T cells, inducing apoptosis and promoting immune tolerance to the transplanted islets [141]. Preclinical studies in immunocompetent diabetic mice demonstrated successful IsletRx engraftment, substantial C-peptide release, and improved glycemic control over three months. Importantly, the iTOL-100 microgel did not impair IsletRx function [142]. Similar results were observed with SA-FasL microgel in islet transplantation in the omentum, further supporting iTOL-100’s potential to enhance immune tolerance [143]. While these findings are promising, the technology remains in the preclinical stage and requires further validation in non-human primates and human patients.

### SIG-002 (Sigilon Therapeutics and Eli Lilly)

SIG-002, developed by Sigilon Therapeutics in collaboration with Eli Lilly, is a novel device designed to encapsulate iPSC-derived pancreatic islets using Afibromer, a chemically modified alginate biomaterial. Afibromer helps protect against immune rejection and fibrosis, both of which can compromise encapsulated stem cells (see discussion on encapsulation technology). In preclinical studies with STZ-induced diabetic mice, SIG-002 promoted iPSC differentiation into insulin-secreting beta cells, maintaining glycemic control for up to 17 weeks. The Afibromer capsule also reduced fibrosis by preventing macrophage attachment, further supporting the device’s efficacy [144, 145]. Despite promising preclinical results, Sigilon’s technology faced setbacks, including the termination of clinical trials for SIG-001 in Hemophilia A and the FDA’s withdrawal of SIG-005 for MPS-1. However, Eli Lilly acquired Sigilon Therapeutics to advance SIG-002. As of now, no clinical trials are underway to assess SIG-002’s efficacy in T1DM patients [146].

## T1DM stem cell therapies: Challenges to clinical translation

**Figure 4.**
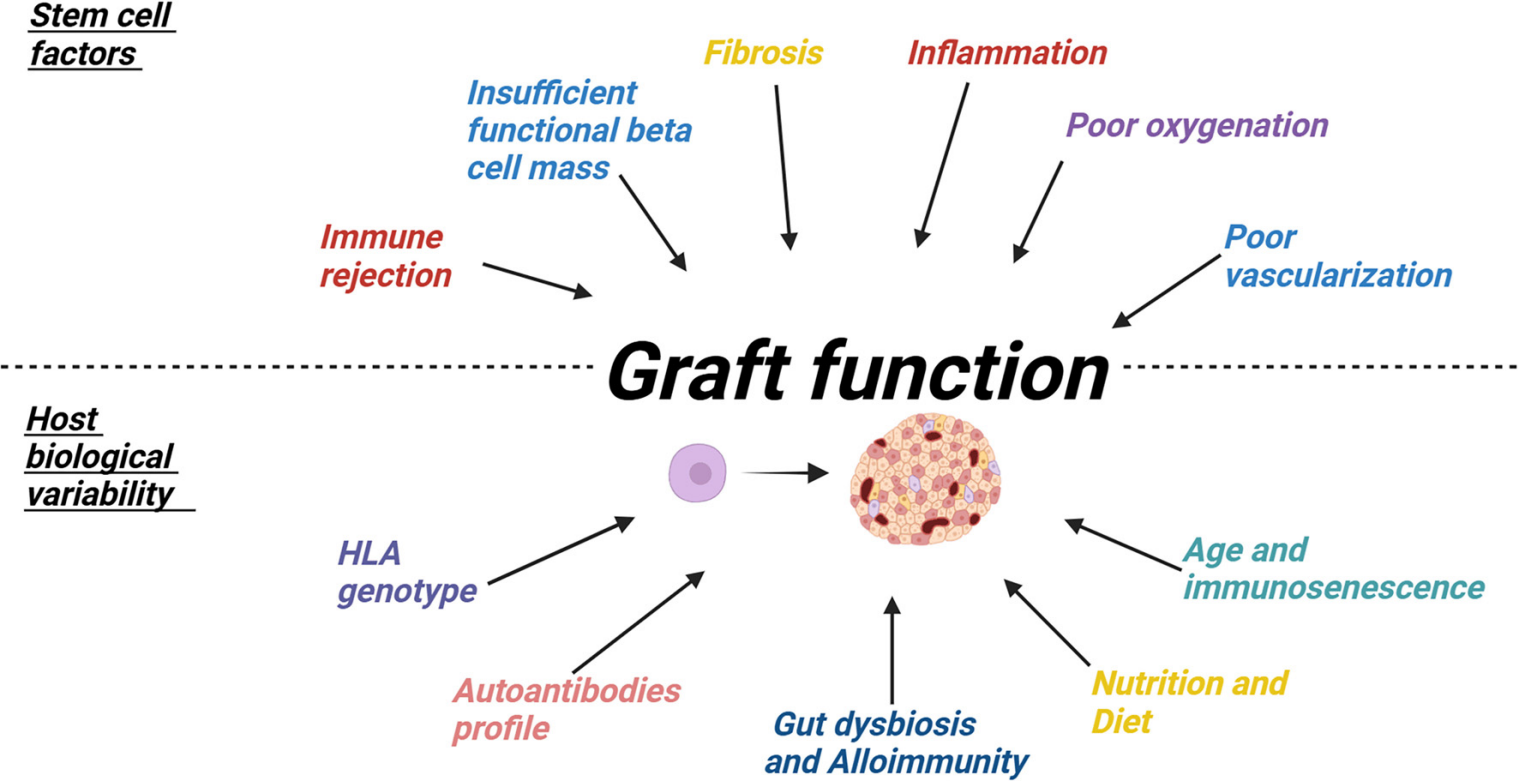
**A summary of key factors that contribute to graft acceptance and function.** Once implanted, stem cells can differentiate into pancreatic islets containing insulin-producing beta cells. However, the success of the graft depends on both intrinsic issues within the graft itself and biological variability among individuals with T1DM. Potential challenges include immune rejection, inflammation, reduced functional beta cell mass, fibrosis, poor oxygenation, and inadequate vascularization—either due to the stem cells or the implanted device. Additionally, biological differences among T1DM patients, such as HLA genotype, autoantibody profile, gut dysbiosis, alloimmunity, nutrition, age, and immunosenescence, can influence graft acceptance and function. Addressing these factors may improve the effectiveness of stem cell therapy for T1DM. Abbreviation: T1DM: Type 1 diabetes mellitus.

Despite significant progress in stem cell-based therapies for T1DM, several challenges continue to hinder their clinical translation (see Figure 4). One major issue is the genetic modification of cells, such as through CRISPR/Cas9, which can lead to off-target mutations and genomic instability, potentially compromising both therapeutic success and patient safety [147]. These unintended genetic alterations may disrupt vital genes, cause chromosomal rearrangements, or introduce unpredictable changes—each posing significant risks for long-term treatment outcomes [107]. Additionally, residual pluripotency in undifferentiated cells raises concerns about teratogenesis and uncontrolled cell division, underscoring the need for continuous monitoring [148]. Ensuring the safety of stem cell-based therapies requires rigorous oversight to mitigate risks such as tumorigenicity or uncontrolled proliferation over the long term. Regulatory agencies must establish clear guidelines for their clinical use, including protocols for handling genetically modified stem cells, long-term monitoring of clinical outcomes, and addressing broader ethical considerations. Successfully navigating these regulatory and ethical challenges will be essential for gaining public trust and ensuring the safe and effective implementation of stem cell therapies for T1DM. Even if functional beta cells are successfully generated, their longevity and viability remain major hurdles. These cells are susceptible to immune rejection, insufficient vascularization, and metabolic stress, all of which may shorten their functional lifespan [149]. Additionally, pancreatic progenitor cells often differentiate into alpha cells rather than insulin-producing beta cells, limiting insulin production [150]. To address this, researchers are exploring strategies to transdifferentiate alpha cells into functional beta cells to increase beta cell mass and improve therapeutic outcomes [151]. However, stem cell–derived beta cells generated through chemical reprogramming are not physiologically or transcriptionally identical to primary human beta cells [152], and the long-term autoimmune and functional consequences of transplanting such cells remain unknown. Immune rejection remains one of the most significant obstacles to stem cell therapy for T1DM. While immunosuppressive treatments can prevent graft rejection, they also increase susceptibility to infections [153]. Clinicians must carefully balance these risks by monitoring for infections, adjusting immunosuppressive regimens as needed, and considering prophylactic treatments to reduce infection risk while ensuring graft survival through personalized management strategies [154]. Scalability is another critical challenge, as stem cell therapies typically require individualized cell processing for each patient—an approach that is resource-intensive and not well-suited for large-scale implementation. Achieving scalability will require advances in cryopreservation and large-scale stem cell culture systems that ensure the long-term viability of stem cell therapies, but these technologies are costly and require significant infrastructure investments [155, 156]. Furthermore, the high costs associated with differentiation, transplantation, and post-transplant monitoring may make these therapies inaccessible to many T1DM patients and financially burdensome for healthcare systems and insurers [157, 158]. The need for specialized facilities and trained healthcare professionals to handle genetically modified cells further limits widespread availability, restricting access to a few select medical centers. Developing cost-effective production methods and establishing dedicated stem cell manufacturing infrastructure within the healthcare system will be critical to making these therapies more broadly accessible.

In addition to the inherent limitations of stem cell therapies, individual variability among T1DM patients complicates graft outcomes. These patients exhibit substantial biological differences, particularly in genetics, immune system activity, gut microbiota, and nutrition, all of which can influence graft survival. For instance, certain HLA genotypes, such as DR3-DQ2 and DR4-DQ8, increase the risk of an aggressive immune response against implanted stem cells [159]. These genetic variations affect T cell activity, making some patients more prone to a stronger T cell-mediated attack on the graft [160]. Another source of variability is the presence of autoantibodies targeting beta cells. Different T1DM patients produce distinct autoantibody profiles, which can influence graft rejection. The number of autoantibodies—such as glutamic acid decarboxylase (GADA), insulinoma antigen-2 (IA-2A), and zinc transporter 8 (ZnT8A)—is now believed to correlate with disease severity, with a more severe autoantibody profile potentially increasing the risk of graft failure [161]. Gut microbiota imbalance (dysbiosis) has also been linked to T1DM and other autoimmune diseases. After transplantation, gut microbiota composition can be significantly affected by diet and immunosuppressive regimens, leading to variations in graft outcomes even among patients undergoing the same stem cell procedure [162]. Gut microbiota can influence alloimmunity, either enhancing or reducing the likelihood of graft rejection. Additionally, the microbiome can metabolize immunosuppressants, causing interpersonal differences in drug effectiveness [163]. For example, gut bacteria can alter the pharmacokinetics and bioavailability of tacrolimus, a commonly used immunosuppressant, which may further complicate graft outcomes [164]. Other factors, such as age and nutrition, also play a crucial role in graft survival. Older patients generally experience better graft outcomes due to a naturally weakened immune response [165]. Similarly, nutritional status is closely tied to graft success, with malnutrition being associated with poorer outcomes [166]. These complexities underscore the need for ongoing research to refine stem cell differentiation protocols, mitigate immune rejection, and better understand patient-specific factors that influence graft success.

## Perspectives

Stem cell therapy T1DM is advancing rapidly, offering promising treatment options, as demonstrated in a single female patient who received chemically reprogrammed autologous stem cells [27]. However, this approach requires complex protocols for genetic manipulation, formulation, and patient-specific administration. While its success in one patient is encouraging, widespread adoption would demand significant resources, making scalability and cost major challenges. SCET is another promising approach that modulates the autoimmune response to preserve pancreatic beta cells, particularly in early-onset T1DM patients with residual function. Its efficacy is greater in patients with preserved beta cell activity, but its therapeutic window is narrow, as newly diagnosed individuals typically retain only 20%–30% of their beta cells [167]. While SCET may benefit early-stage T1DM, it is less suitable for advanced disease, emphasizing the need for alternative therapies [168]. Among current stem cell-based treatments, Vertex’s VX-880 appears closest to regulatory approval. Ongoing Phase 1/2/3 trials have shown promising outcomes, including insulin independence in some patients. However, challenges remain, including scalability, the need for immunosuppression, and long-term safety. Other therapies—such as SR-02, and CRISPR-based approaches (VCTX211 and VCTX210A)—are progressing through clinical trials but require further validation. A key issue in evaluating stem cell therapies is defining “insulin independence.” Many clinical trials classify patients as insulin-independent if they cease exogenous insulin use, but this definition lacks rigor. For example, Vertex Pharmaceuticals only considers patients who remain off insulin for over a year as insulin-independent [127, 169]. Stricter criteria are needed to ensure accurate assessment and prevent premature optimism. To enhance the effectiveness of stem cell therapies, combining them with adjunct treatments like immunotherapy may offer synergistic benefits. Strategies such as Teplizumab or regulatory T-cell-based therapies [170] could improve immune tolerance, potentially increasing the success of stem cell-based treatments. Integrating these approaches may address immune system challenges and improve long-term outcomes. Encapsulation technologies, despite advancing to clinical trials, still face hurdles, particularly immune rejection and device durability (summarized in Table 1 and Figure 2). The failure of ViaCyte’s PEC-Encap highlights the limitations of encapsulation alone in sustaining long-term stem cell survival and function for T1DM treatment. A promising alternative is hypoimmune stem cells, genetically engineered to evade immune detection. Advances in CRISPR-based immune editing, such as Sana Biotechnology’s (HIP), demonstrate the potential for stem cell therapies without lifelong immunosuppression or encapsulation barriers, addressing the root cause of immune rejection.

While preclinical studies in humanized mice and non-human primates have demonstrated the feasibility of hypoimmune beta-cell grafts in sustaining insulin secretion and glycemic control, these approaches remain experimental. Long-term safety, functional durability, and clinical efficacy in humans have yet to be established, and unforeseen immune responses or tumorigenicity risks may arise. Despite these uncertainties, rapid advancements in immune engineering suggest that hypoimmune strategies could offer a more scalable and effective alternative to encapsulation. However, clinical validation remains essential before they can be considered a definitive solution for T1DM treatment. Based on the current evidence, we believe hypoimmune strategies will likely become the preferred approach in future stem cell-based therapies for T1DM. It is important to note that stem cell therapies for T1DM remain experimental. While FDA-approved treatments such as immunotherapy and islet transplantation are the current standard, stem cell therapies hold significant promise. Clinicians may consider referring eligible patients, particularly those already on immunosuppression, to ongoing clinical trials as the field continues to evolve.

## Conclusion

In conclusion, stem cell therapy holds immense potential as a long-term treatment for T1DM. Collaborative efforts between researchers and the biotech industry have accelerated progress, bringing these therapies closer to clinical application. However, regulatory approval pathways and long-term safety monitoring protocols must evolve alongside these innovations—particularly for gene-edited hypoimmune stem cells—to ensure both safety and public trust. While significant challenges remain, including immune compatibility and graft durability, advancements in stem cell research offer a promising future. Notably, the development of hypoimmune stem cell therapies represents a major milestone in overcoming immune rejection, paving the way for more scalable and effective treatments. Moving forward, rigorous research and large-scale clinical evaluations will be essential to address these challenges and transition stem cell therapies from experimental treatments to viable long-term solutions for T1DM.

**Note added in proofs:** On March 28, 2025, Vertex Pharmaceuticals announced the discontinuation of VX-264 clinical trial as the efficacy data did not result in the required levels of insulin production. Meanwhile, Zimislecel (formerly VX-880) remains in development and is on track to complete enrolment in the first half of 2025, with likely global regulatory submissions in 2026.
